# Nutrient allocation patterns in different aboveground organs at different reproductive stages of four introduced *Calligonum* species in a common garden in northwestern China

**DOI:** 10.3389/fpls.2024.1504216

**Published:** 2024-12-12

**Authors:** Ji-Yuan Liu, Xue-Lian Zhang, Xin-Yue Jin, Meng-Ting Wang, Yuan-Yuan Zhang, Xi-Yong Wang

**Affiliations:** ^1^ State Key Laboratory of Desert and Oasis Ecology, Key Laboratory of Ecological Safety and Sustainable Development in Arid Lands, Xinjiang Institute of Ecology and Geography, Chinese Academy of Sciences, Urumqi, Xinjiang, China; ^2^ Key Laboratory of Conservation and Utilization of Plant Gene Resources, Xinjiang Institute of Ecology and Geography, Chinese Academy of Sciences, Urumqi, Xinjiang, China; ^3^ Anhui Province Key Laboratory of the Biodiversity Study and Ecology Conservation in Southwest Anhui, College of Life Sciences, Anqing Normal University, Anqing, Anhui, China; ^4^ Turpan Desert Botanical Garden, Chinese Academy of Sciences, Turpan, Xinjiang, China

**Keywords:** stoichiometry, *Calligonum*, reproductive period, organ, common garden, shrub with assimilative branch

## Abstract

**Introduction:**

The *Calligonum* species is a typical shrub with assimilative branches (ABs) in arid regions in Central Asia. The nutrient distribution patterns at different reproductive stages are of great significance for further understanding the ecological adaptation and survival strategies of plants.

**Methods:**

In the present study, a common garden experiment was employed to avoid interference by environmental heterogeneity. Furthermore, the nitrogen (N), phosphorus (P), and potassium (K) allocation characteristics in the supporting organs (mature branches), photosynthetic organs (ABs), and reproductive organs (flowers and fruits) of *Calligonum caput-medusae* (CC), *Calligonum arborescens* (CA), *Calligonum rubicundum* (CR), and *Calligonum klementzii* (CK) during the flowering, unripe fruit, and ripe fruit phases were systematically analyzed.

**Results:**

Aboveground organs were the main factors affecting the variation of N, P, and K concentrations and their stoichiometric ratios, and the reproductive stages were secondary factors affecting N, P, and the P:K ratio and species were secondary factors affecting K and the N:P and N:K ratios. Meanwhile, significant interactions were found for all three of the aforementioned factors. The N and P concentrations in the ABs of the four species were highest during the flowering phase, while the N:P ratio was lowest, which then gradually decreased and increased, respectively, during plant growth. This result supported the growth rate hypothesis, i.e., that the growth rate is highest during the early growth stage. In the growth period, the N, P, and K concentrations in each organ of the four *Calligonum* species followed the power law, with the allocation rates of N and P being generally higher than K. There were differences among the species as the N−P scaling exponent in the ABs of CR was only 0.256; according to the scaling exponent law, this species was the least stressed and had the strongest environmental adaptability. Overall, the adaptability of the four species could be ranked as CR > CA > CC > CK. In conclusion, there were significant differences in nutrient traits among different aboveground organs, species, and reproductive stages.

**Discussion:**

The results of this study contribute to a deeper understanding of the nutrient allocation strategies of different *Calligonum* species and provide scientific evidence for the *ex-situ* conservation and fixation application of these species.

## 1 Introduction

Nitrogen (N), phosphorus (P), and potassium (K) are essential elements for plant growth and development, playing crucial roles in various physiological regulatory mechanisms (Fan et al., 2015). Ecological stoichiometry theory simplifies complex ecological processes by studying the quantitative relationships and dynamic balances between fundamental elemental compositions (Elser et al., 2011; James Elser, 2006). Research on plant stoichiometric characteristics in different habitats often shows spatial heterogeneity (Wang et al., 2024), making it challenging to explain the effects of plant growth conditions. Plant nutrient concentrations vary across different growth stages and among aboveground organs in response to photosynthetic efficiency and nutrient demand, aiding in maintaining plant functions and environmental adaptability (Li et al., 2017; Wright and Westoby, 2003; Yang and Luo, 2011). Leaves accumulate photosynthetic products through photosynthesis (Tang et al., 2018); stems provide transport pathways and connections between aboveground organs; and reproductive organs serve as storage structures for assimilates (Zhao et al., 2016; Wang et al., 2019). Numerous studies have shown that the nutrient characteristics of leaves and stems exhibit covariation at the individual level (Touati et al., 2022; Zhang et al., 2018), with significant interspecific or functional group differences, laying the foundation for exploring differential nutrient utilization strategies across species. In the arid regions of Central Asia, some shrubs, in adapting to the extreme arid environment, have reduced or lost leaves, replacing them with specialized green assimilative branches (ABs) for photosynthesis (Meng et al., 2022). ABs are annual organs, with reproductive organs attached to nodes. Typically, some ABs lignify to form mature branches toward the end of the growing season, providing structural support for continuous growth. Studies indicate that ABs exhibit distinct morphological and physiological ecological traits compared to other plant types. For instance, C_3_ plants, especially C_3_ shrubs with ABs, have significantly lower P concentrations than non-AB C_3_ plants, herbs, and other shrubs (Tao et al., 2016). These shrubs also have lower N concentrations, suggesting that desert plants in extreme environments develop specific stoichiometric characteristics based on their life history strategies (Tao et al., 2016). Despite convergent morphological traits in different species with ABs (Meng et al., 2022), the trait relationships and environmental variation patterns of nutrients often show significant interspecies differences (Meng et al., 2023). Central Asia is a key area of origin for shrubs with ABs in temperate deserts worldwide. As such, exploring the ecological adaptability and survival strategies of shrubs with ABs not only helps reveal their evolutionary trends but also provides theoretical support for the restoration and sustainable management of degraded desert ecosystems.

Species of the genus *Calligonum* (Polygonaceae) are the typical shrubs with ABs, and they are widely distributed in the arid regions of Central Asia, extending to West Asia and even northern Africa. China hosts the highest diversity of *Calligonum* species, with 23 species. These species are excellent pioneer plants for sand fixation and ecological restoration (Wang et al., 2016). *Calligonum* species have “Z”-shaped branches with short internodes; leaves reduced to very short remnants, membranous sheaths (Wang et al., 2016), or entirely reduced to small stalks; and their ABs have strong photosynthetic capabilities (Liu et al., 2016). Given the ecological and scientific value of *Calligonum*, research has been conducted on the taxonomy, reproductive characteristics, morphological structure (Liu et al., 2014), genetic diversity, physiological responses to environmental stress (Liu et al., 2011), and stoichiometric characteristics of several *Calligonum* species (Meng et al., 2023). Additionally, *Calligonum* species have been extensively used in the restoration of degraded ecosystems and the construction of fixation systems in the arid regions in Central Asia, yielding significant ecological benefits. Therefore, we aim to address the scientific question of whether the ecological adaptability and nutrient strategies of different *Calligonum* species change after being planted in the same habitat and adapting for a period of time.

A common garden experiment can minimize the environmental heterogeneity affecting the growth of an introduced species, providing an excellent platform for long-term observation and analysis of the short-term ecological plasticity and long-term adaptive evolution of different species. This study focuses on the extremely arid and hot environment of the Turpan Desert Botanical Garden, using four introduced *Calligonum* species [*Calligonum caput-medusae* (CC), *Calligonum arborescens* (CA), *Calligonum rubicundum* (CR), and *Calligonum klementzii* (CK)] as the research subjects. By discussing the stoichiometric characteristics of N, P, and K during three reproductive phases [supporting organs (mature branches), photosynthetic organs (Abs), and reproductive organs (flowers and fruits)], we aim to explore the variability and determine if there is convergence among species. We will test the growth rate hypothesis, the N:P threshold hypothesis, and the power law of N−P stoichiometry. Given the aforementioned summary, we hypothesize that, if interspecific differences exist, it will confirm the species composition hypothesis, indicating that different species will not form similar nutrient patterns after a period of co-adaptation. The results of this study will help to further understand the nutrient strategies of ABs in arid regions and their adaptive strategies under the same extreme conditions, providing a theoretical basis for the ecological protection and management of arid regions.

## 2 Materials and methods

### 2.1 Study area

The Turpan Desert Botanical Garden (89°11′ E, 40°51′ N; alt. −105 m–−76 m) is located in the southeastern sandy dessert of the Turpan Basin in Xinjiang, China. It is the lowest-altitude botanical garden in the world, covering an area of 150 hm^2^. The botanical garden is located in the heart of the Central Asian arid region, within the Eurasian continental interior, and has a temperate inland arid desert climate. The area receives an average annual precipitation of only 16.4 mm, with an annual evaporation as high as 3000.0 mm. It has abundant sunshine, with an annual total of 3049.5 h of sunlight and a total annual radiation of 139.5 kcal cm^−2^. The annual average temperature is 13.9°C, with extreme lows of −28°C and extreme highs reaching up to 49.6°C. In summer, the surface temperature of the sand exceeds 80°C. The area frequently experiences strong winds, with an annual average of 26 days of gale-force winds (≥ level 8), peaking at 68 days. The annual cumulative temperature (≥ 10°C) averages 5454.8°C, and the frost-free period averages 265.6 d. The zonal soil is primitive gray-brown desert soil with a pH range of 8.6–9.1. The groundwater depth ranges from 10.0 m to 15.0 m (Duan and Shi, 2019). For detailed information on the climatic data for April, May, and June in the research garden, see the **Supplementary Materials** (**Supplementary Table S2**). The Turpan Desert Botanical Garden has become a reserve of central Asian desert plant resources (species and genes), conserving over 800 species of desert plants. These species belong to 87 families and 385 genera, covering the main taxa of central Asian desert flora. Among them, there are 43 rare and endemic desert plant species, including 21 endemic species and 4 relict species. The Turpan Desert Botanical Garden is not only an ideal site for studying plant adaptability and survival strategies in extreme global environments but also provides valuable biological resources for exploring plant adaptation mechanisms to extreme drought and high-temperature conditions.

### 2.2 Sample collection

**Figure 1:**
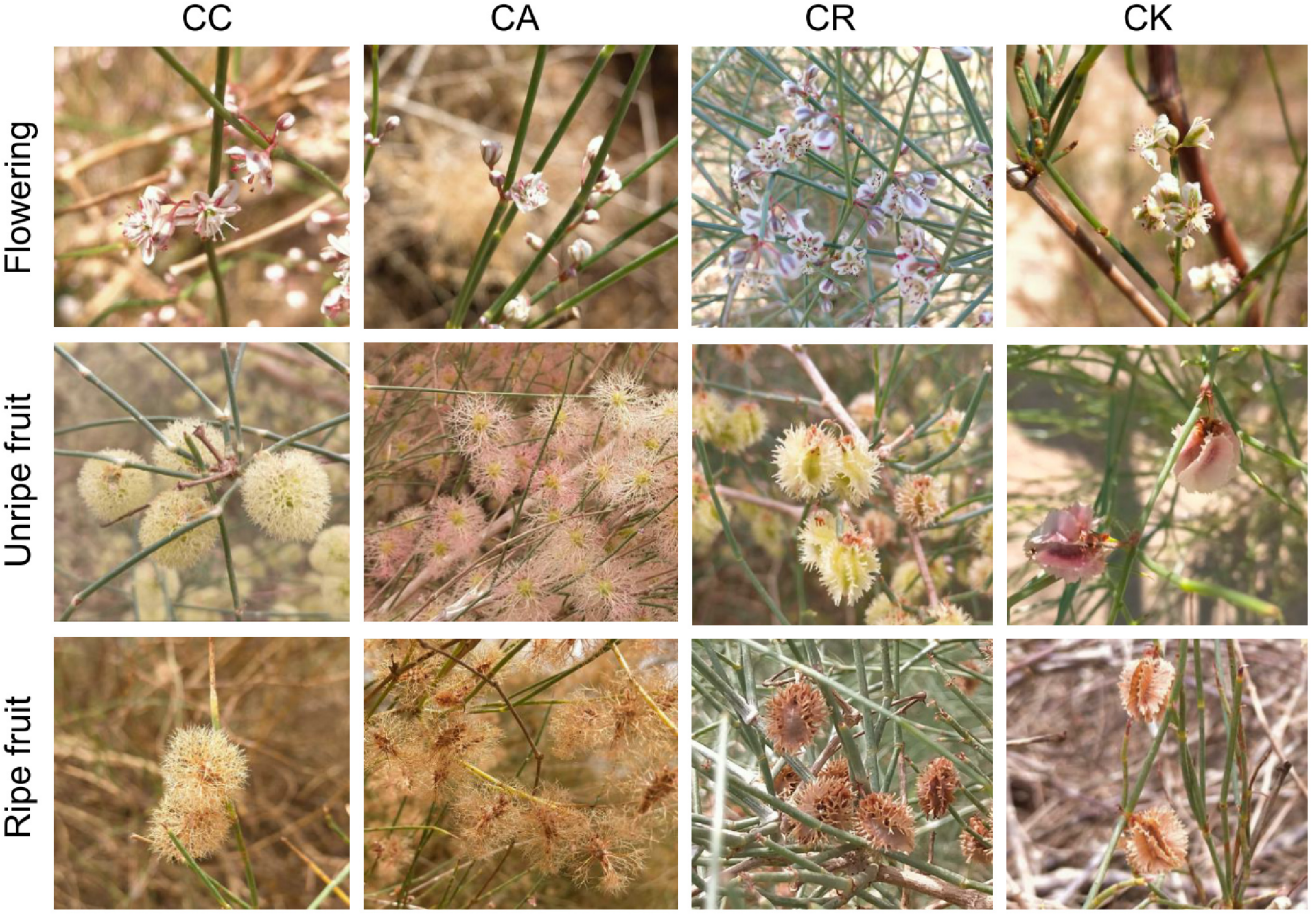
Photographs of the four *Calligonum* species at different reproductive stages. CC, CA, CR, and CK refer to *Calligonum caput-medusae*, *Calligonum arborescens*, *Calligonum rubicundum*, and *Calligonum klementzii*, respectively. CC: plant height 2.0 m–4.0 m, crown width 1.5 m–2.8 m; CA: plant height 2.2 m–4.5 m, crown width 1.5 m–3.3 m; CR: plant height 0.9 m–1.7 m, crown width: 0.9 m–2.1 m; CK: plant height: 0.8 m–1.5 m, crown width: 0.9 m–1.8 m.

This study selected four *Calligonum* species (i.e., CC, CA, CR, and CK) as subjects. Samples of reproductive organs, green ABs, and mature branches were collected at different reproductive stages (**Figure 1**). CC and CA originated from Repetek in the eastern Karakum Desert, Turkmenistan, and were planted in 1985 through seed germination. CR and CK are native to Xinjiang, China (but not found in the Turpan region), with seed sources from Burqin County, Altay Prefecture, and Jimusar County, Changji Prefecture, respectively. They were introduced to the botanical garden in 1982 and 2006. The four species of *Calligonum* originated from regions that are geographically distant from each other, indicating significant geographical isolation. Compared to other *Calligonum* species, these four had a relatively long introduction period, which was helpful for the comparative study of physiological and ecological adaptability between species. This was the main reason for choosing these four species as research subjects. After planting, the*Calligonum* species were irrigated by trench flooding in both summer and winter to ensure survival. From April to June 2021, in the reproductive period for *Calligonum* species, six individuals of similar size (standard trees) were selected and marked for each species in the Turpan Desert Botanical Garden, totaling 24 plants. On 10 April 2021, samples were first collected from the marked plants. For this purpose, 3–5 mature branches (less than 3 years old and with a diameter not exceeding 5 mm) were cut from the north, south, east, and west sides of each plant crown. The samples were quickly placed in sampling bags, stored in a cooler with ice packs, and brought back to the laboratory as soon as possible. The flowers (early reproductive organs), green ABs, and mature branches of each plant were separated and placed in labeled envelopes for drying. On 10 May and 10 June, the samples of three organs were collected using the same method for the unripe fruit phase (URP) and the ripe fruit phase (RFP). In June, some fruits had already fallen, and mature seeds were collected using a combination of collection nets and *in situ* fruit sampling.

### 2.3 Nutrient concentration determination

The plant samples collected in the field were dried at 70°C in an oven, powdered in a vibratory disc mill (MM 400, Retsch GmbH Inc., Haan, Germany), and then stored in zipper bags. The total nitrogen (N) concentration of the samples (mg g^−1^) was calculated using Kjeldahl digestion, the total phosphorus (P; mg g^−1^) using the colorimetric ammonium molybdate method and an AA3 (Auto Analyzer 3, SEAL, Germany), and the total potassium (K) by perchloric acid-concentrated sulfuric acid digestion followed by flame photometry. The results for N, P, and K were expressed in mass concentrations (mg g^−1^), and the stoichiometric ratios (i.e., N:P, N:K, and P:K) were also calculated.

### 2.4 Statistics

The data were checked for normality using the Kolmogorov–Smirnov test, and data that did not fit the normal distribution were logarithmically transformed before being used. A nested ANOVA (analysis of variance) was used to analyze the relative contributions of reproductive stages, species, and organs to the variation in stoichiometric traits among the four *Calligonum* species (Nested Procedure, SAS version 8.0; SAS Institute Inc., Cary, NC, USA) (Liu et al., 2010). By conducting a three-way ANOVA, the effects of different reproductive stages, species, and organs, along with their interactions, on the stoichiometric traits of *Calligonum* species were analyzed. A one-way ANOVA was used to explore the differences in stoichiometric traits of the *Calligonum* species across different reproductive stages, species, and organs. Homogeneity of variance was tested using Levene’s test. Tukey’s HSD test was used for multiple comparisons. The one-way ANOVA was performed using SPSS 26.0. Data were grouped, and summary statistics such as means and standard errors were calculated using the ‘dplyr’ package in R version 4.3.2. The three-way analysis of variance was conducted using the ‘aov’ function in the ‘stats’ package. Graphs were generated using the ‘ggplot2’ package.

The allometric relationships between nutrient elements are represented by the equation *Y = bXa*, where *Y* and *X* are trait indices, *b* is a constant, and *a* is the allometric exponent (Niklas, 2004). Two scenarios may arise: 1) *a* = 1 indicates an anisometric relationship, i.e., the dependent variable and the independent variable change uniformly or in the same proportion; 2) *a* ≠ 1 represents an allometric relationship (*a* > 1 indicates the relationship was hyperallometric and *a* < 1 means it was hypoallometric), where changes between the dependent and independent variables are not uniform or proportional (Zhu et al., 2011). Typically, after logarithmic transformation of the power function, reduced major axis (RMA) linear regression (i.e., Model Type II) is conducted to estimate the scaling slope, a 95% confidence interval (95% CI), and the coefficient of determination (*R* ^2^). Allometric scaling analysis was performed using the SMATR package (Meng et al., 2022).

## 3 Results

### 3.1 Sources of stoichiometric variation in different aboveground organs of the four *Calligonum* species during the reproductive period

**Figure 2:**
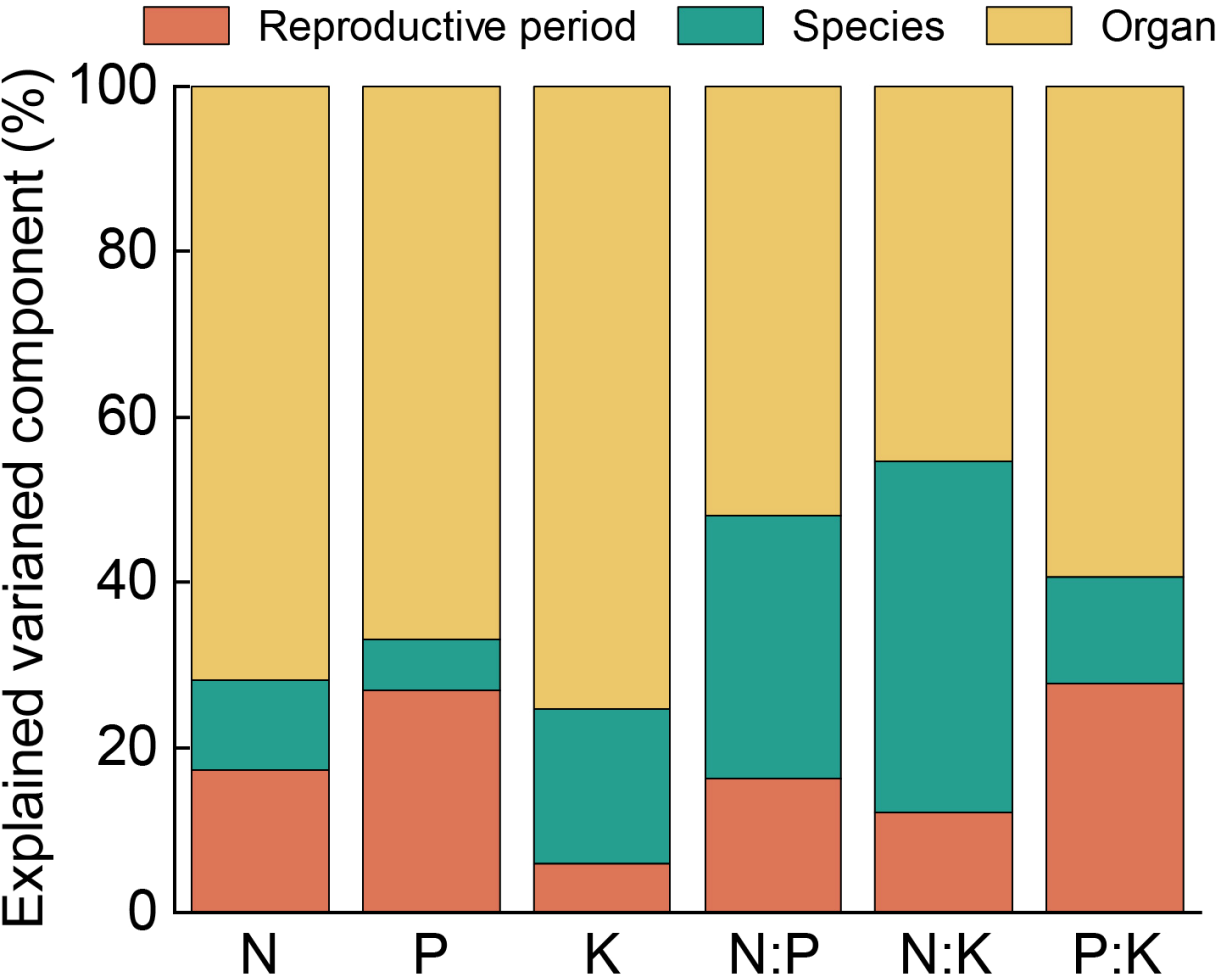
The hierarchical variance analysis of the six stoichiometric traits of the four *Calligonum* species.

Variance component analysis for each variable (**Figure 2**) indicated that the stoichiometric traits exhibited the greatest variation among the organs, all exceeding 45%. The contribution of the organs to the variation of the nutrients (N, P, and K) could be ranked as K > N > P, accounting for 75.41%, 71.92%, and 67.04% of the total variation, respectively. In the different reproductive stages, the variation in K and N:K was minor, comprising less than 15% of the total variation; however, the variation of the other four nutrient traits explained 16.19%–27.65%. The interspecific variation of N, P, and P:K was the smallest, accounting for 10.73%, 5.99%, and 12.94% of the total variation, respectively. The interspecific variation of K, N:P, and N:K ranged from 18.69% to 42.5%. In addition, the results of three-way ANOVA (**Table 1**) showed that the reproductive stage, species, and organs and their interactions had a significant effect on all of the stoichiometric traits of the *Calligonum* species (*P* < 0.001). Based on the *F*-values of the three-way ANOVA, organ type had the greatest impact on the stoichiometric traits of the *Calligonum* species, while the interaction effects of reproductive stage, species, and organs were relatively weaker. These results confirmed that the organs were the primary cause of variation in nutrient traits among the *Calligonum* species.

**Table 1:** Three-way ANOVA of stoichiometric traits in four *Calligonum* species for reproductive phase, species, and organ (*F*-value).

Source	N	P	K	N:P	N:K	P:K
Reproductive phase	565.12***	3387.27***	51.458***	75.563***	93.029***	215.630***
Species	155.50***	430.27***	74.341***	49.194***	183.638***	54.106***
Organ	1802.50***	6332.87***	445.718***	167.918	208.727***	337.527***
Reproductive phase × Species	38.76***	35.79***	17.158***	24.865***	17.751***	7.303***
Reproductive phase × Organ	191.97***	780.43***	41.340***	16.259***	16.838***	27.827***
Species × Organ	21.05***	119.50***	30.970***	6.148***	25.227***	15.154***
Reproductive phase × Species × Organ	15.43***	27.57***	6.061***	3.971***	5.603***	4.299***

***P < 0.001.

### 3.2 Differences in nutrient characteristics of different aboveground organs during the reproductive period of the four *Calligonum* species

**Figure 3:**
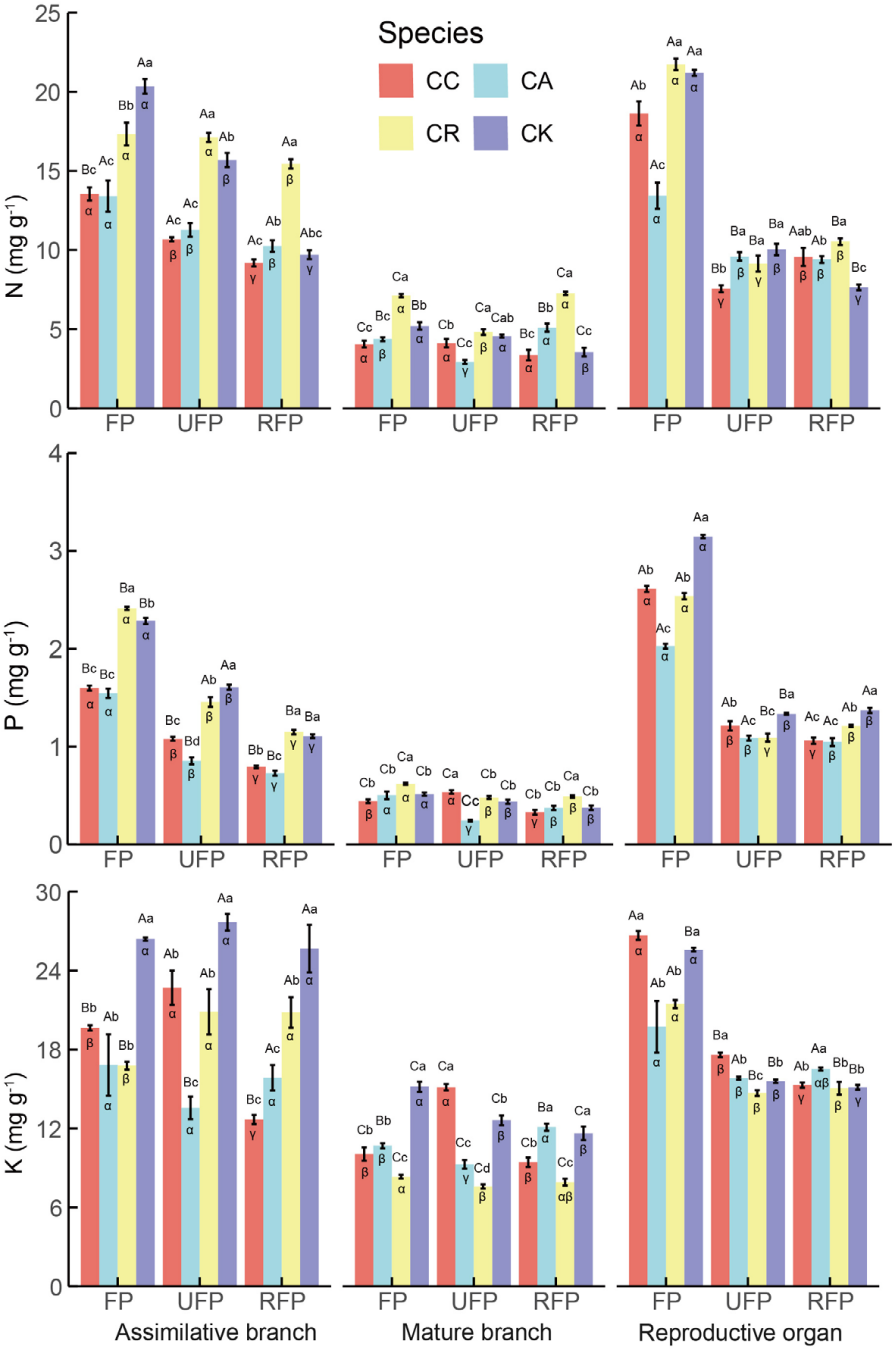
N, P, and, K concentrations in different aboveground organs during the reproductive period of four *Calligonum* species in a common garden. CC, CA, CR, and CK refer to *Calligonum caput-medusae*, *Calligonum arborescens*, *Calligonum rubicundum*, and *Calligonum klementzii*, respectively. FP, UFP, and RFP refer to the flowering phase, unripe fruit phase, and ripe fruit phase, respectively. α, β, and γ: markers for differences between different reproductive periods for the same organ and the same species. A, B, and C markers for differences between different organs within the same species and the same period. a, b, and c markers for significant differences between the same organs in different species within the same period.

Significant differences in N, P, and K concentrations in the *Calligonum* species, reproductive stages, and organs (**Figure 3**) were detected. The stoichiometric traits exhibited the greatest variability among the organs, so the initial focus of this study was on the differences in N, P, and K concentrations in the aboveground organs. Overall, across all reproductive stages, the N, P, and K concentrations in different aboveground organs of the four *Calligonum* species were significantly lower in the mature branches. In the flowering period, except for CK, where K was highest in the ABs with 26.399 mg g^−1^, the N, P, and K concentrations were significantly higher in the reproductive organs of the *Calligonum* species (CC, CA, CR). In this period, the average concentrations of N, P, and K in the reproductive organs of the four species were 18.746 mg g^−1^, 2.581 mg g^−1^, and 26.368 mg g^−1^, respectively. Furthermore, in the ripe fruit phase, the P concentration across different aboveground organs of all four species showed a consistent pattern: reproductive organs > ABs > mature branches.

Specifically, the N and P concentrations in the ABs of the four species showed a consistent pattern through the reproductive stages: flowering phase (FP) > unripe-fruit phase > ripe-fruit phase. For the N concentrations in the ABs of the different species, CK was significantly highest in the FP at 20.338 mg g^−1^, and CR was significantly highest in the unripe fruit phase and ripe fruit phase at 17.119 mg g^−1^ and 15.447 mg g^−1^, respectively. For the P concentration in the ABs, CR, and CK were significantly higher than the other two species in all reproductive stages. CK had the highest K concentration in the ABs across all reproductive stages, with an average of 26.581 mg g^−1^, and there was no significant difference in K concentration across the different stages for CK.

When comparing the concentrations in the reproductive organs, all four *Calligonum* species showed a similar pattern throughout the reproductive stages: N, P, and K were significantly higher in the FP, decreased significantly (by approximately 20%–60%) in the unripe fruit phase, and showed no significant changes between the unripe fruit phase and the ripe fruit phase. In the flowering phase, the reproductive organs of CA had the lowest N, P, and K concentrations of 13.429 mg g^−1^, 2.026 mg g^−1^, and 19.737 mg g^−1^, respectively. In addition, the concentrations in the mature branches of the four species remained relatively stable across the different reproductive stages, and there were no significant fluctuations.

In summary, the N and P concentrations in the ABs and reproductive organs of the four species were generally highest in the FP, which may be related to the high demand for key nutrients in the early stage of reproduction. In addition, the K concentration in the ABs of CK was significantly higher than that of the other species across all reproductive stages, reflecting its unique adaptation and nutrient utilization strategy. However, the differences in nutrient concentrations between species or organs in the late reproductive phase decreased; thus, *Calligonum* species may tend to a more unified nutrient allocation pattern to optimize resource utilization and survival strategies. The formation of this pattern may be an adaptation to the stresses of extreme arid environments by adjusting nutrient allocation to improve reproductive success and population survival.

**Figure 4:**
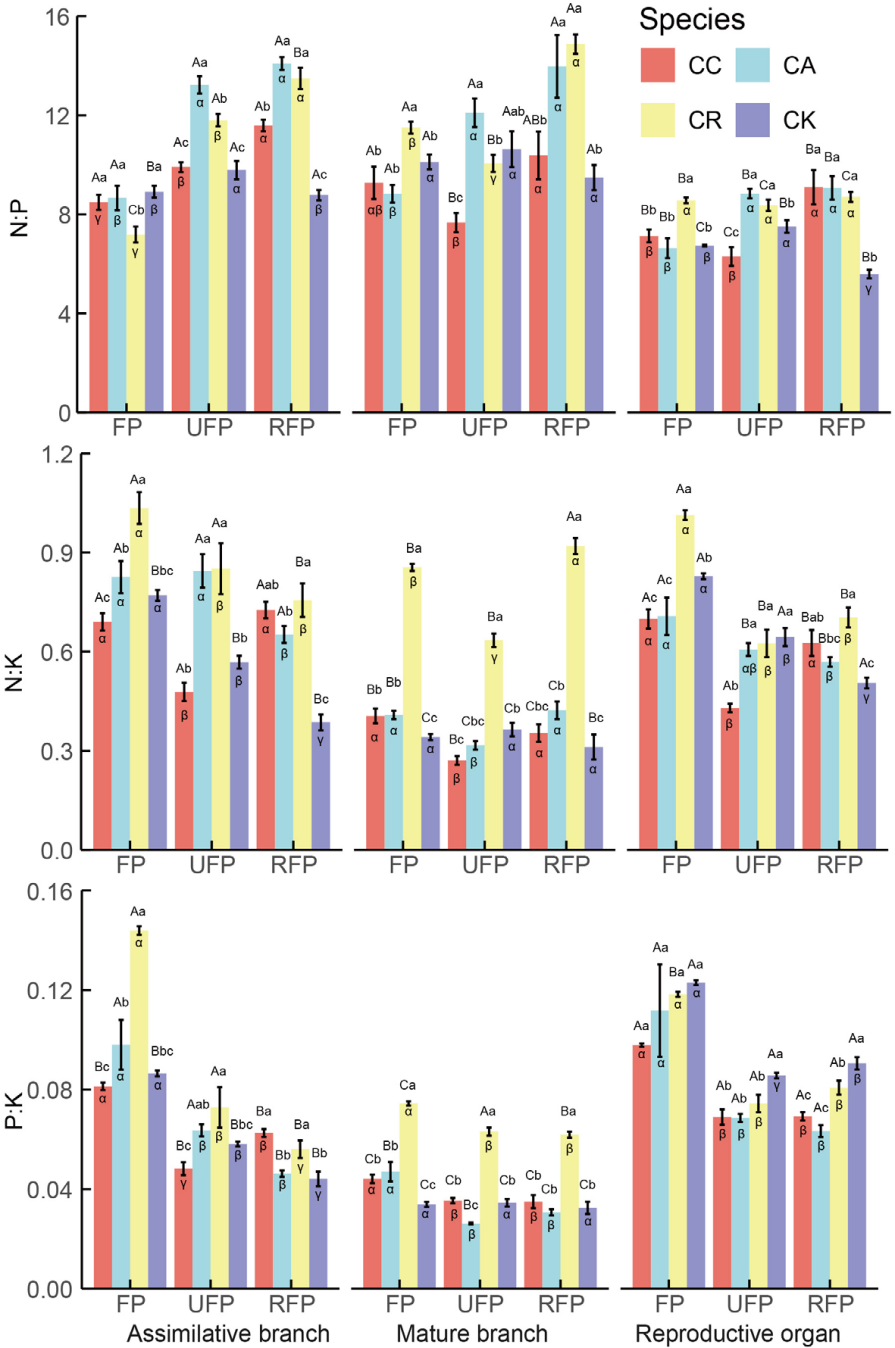
Stoichiometric ratios in different aboveground organs during the reproductive period of four *Calligonum* species in a common garden. CC, CA, CR, and CK refer to *Calligonum caput-medusae*, *Calligonum arborescens*, *Calligonum rubicundum*, and *Calligonum klementzii*, respectively. FP, UFP, and RFP refer to the flowering phase, unripe fruit phase, and ripe fruit phase, respectively. α, β, and γ: markers for differences between different reproductive periods for the same organ and the same species. A, B, and C markers for differences between different organs within the same species and the same period. a, b, and c markers for significant differences between the same organs in different species within the same period.

Significant differences existed in the stoichiometric ratios among the different reproductive stages, species, and organs of the *Calligonum* species (**Figure 4**). In the reproductive period, the N:P in the ABs of the four *Calligonum* species, except for the CA at the ripe fruit phase (14.09), which fell within the range of 14–16, were all below 14. Across all reproductive stages, the reproductive organs exhibited the lowest N:P. In terms of ABs at different reproductive stages, except for the ABs of CK, which showed a trend of increasing first and then decreasing, the other three species showed significantly lower N:P in the flowering phase (an average of 8.112) and as the reproductive process progressed, there was a significant increase in the N:P ratio by the ripe-fruit phase, with the ABs of these three species averaging an N:P of 13.056. Among the different species, the N:P ratio in the aboveground organs of CA and CR during each reproductive stage was consistently higher. Notably, the N:P ratio in the reproductive organs of CK was significantly lower across all reproductive stages. At various reproductive stages, the N:K ratio in the ABs of all four *Calligonum* species was consistently < 2.1, while K:P > 3.4. The N:K and P:K ratios in the mature branches were significantly lower than in other organs. Across the different reproductive stages, the N:K ratio in the ABs of CR and CK followed the pattern of flowering phase > unripe fruit phase > ripe fruit phase. The P:K ratio in the ABs, except for CC which showed a decrease followed by an increase, exhibited a decreasing trend as reproduction progressed. Moreover, the N:K ratio in mature branches, except for CK (average 0.339), showed no significant differences throughout the reproductive stages. The other three species exhibited significantly lower N:K during the unripe fruit phase. The N:K and P:K ratios in reproductive organs were highest during the flowering phase, averaging 0.812 and 0.114, respectively, and decreased significantly during the ripe fruit phase and unripe fruit phase, stabilizing thereafter. Among different species, the N:K and P:K ratios in the ABs and mature branches were highest in CR across all reproductive stages. The results indicated significant differences and stage-dependence in the nutrient ratios of various aboveground organs during the reproductive period. In this study, the N:P ratio in the ABs was generally low, below 14, and was even lower in reproductive organs, highlighting the high demand for P during critical reproductive phases. Moreover, with propagation, the N:P ratio in the ABs showed a significant upward trend. In addition, the N:K and P:K ratios in the ABs were the highest in the flowering phase and then gradually decreased in the unripe fruit phase and the ripe fruit phase, which may reflect a change in nutrient requirements in the growth and development stages. The N:K and P:K ratios in mature branches decreased significantly in the unripe fruit phase. Overall, these results emphasize the importance of dynamic nutrient management across different growth stages and organs.

### 3.3 Nutrient covariation in different aboveground organs during the reproductive period of the four *Calligonum* species

**Figure 5:**
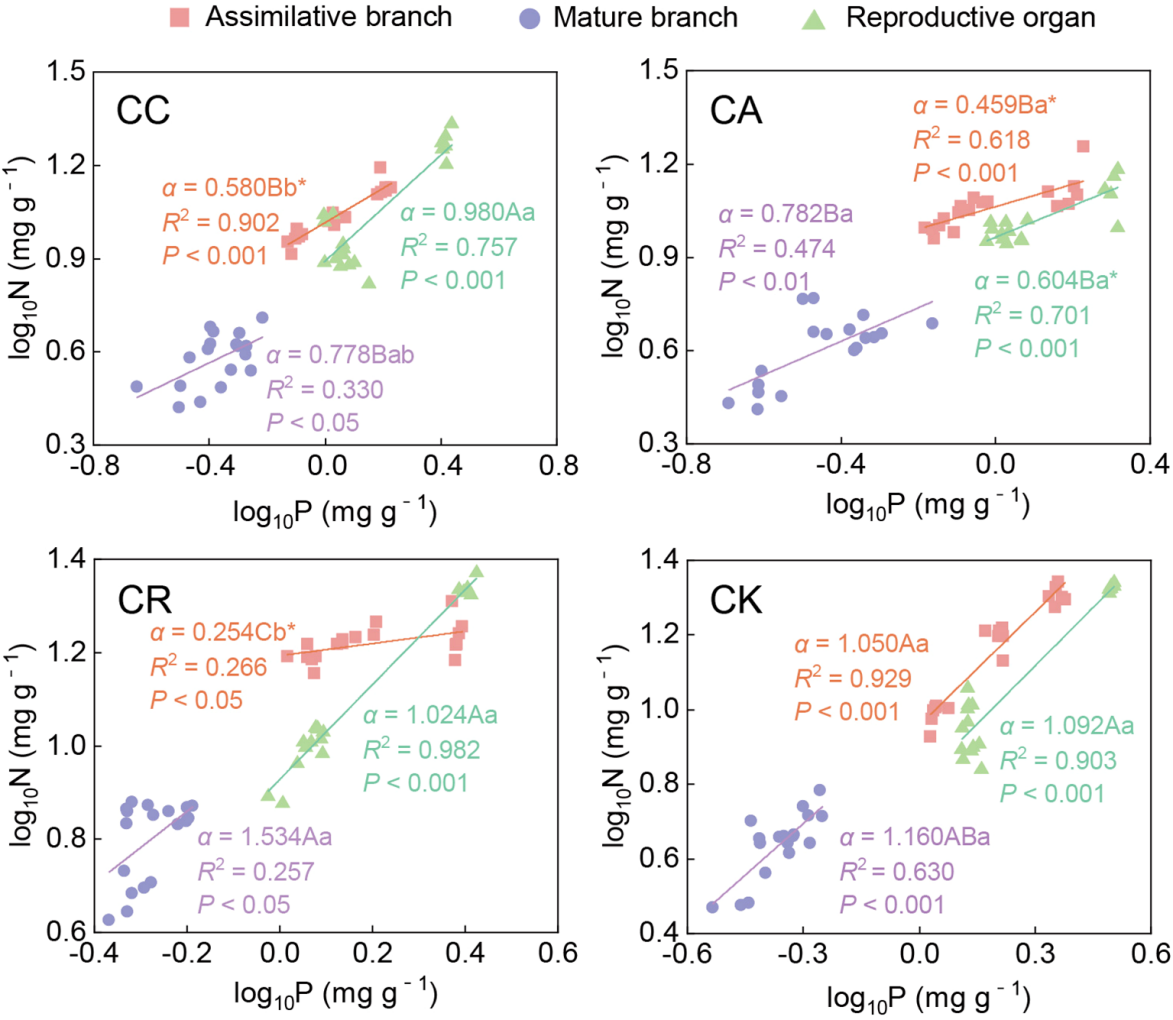
Allometric relationship between N and P in different aboveground organs of four *Calligonum* species. A, B, and C markers for differences between different species for the same organ. a, b, and c markers for differences between different organs of the same species. * marker for significant difference from the assumed slope of 1 (*P* < 0.05).

According to the results of the allometric relationship analysis (**Figure 5**; **Supplementary Table S1**), 31 of the 36 stoichiometric pairs of different aboveground organs of the four species reached a significant level (*P* < 0.05). For these 31 pairs, the scaling exponents of 15 stoichiometric pairs did not significantly differ from 1.0 (i.e., isometric). Among the 16 non-isometric relationships, only 5 were hypoallometric, while most (10) of them (11 pairs) were hyperallometric. In addition, only one stoichiometric pair showed that the scaling exponent was less than −1. The N–P allometric relationship in different aboveground organs of the four *Calligonum* species showed significant interspecific and inter-organ differences. Among the four species, the N–P scaling exponents in the ABs showed that only CK (1.050) did not significantly differ while the other three species showed hypoallometric relationships, and CR had the lowest scaling exponent (0.254). There was no significant difference in the N–P scaling exponents with 1 in mature branches of the four species, and CR had the highest scaling exponent at 1.534, significantly higher than the other three species. In the reproductive organs, only CA (0.604) showed a significant hypoallometric relationship, while the other three species were isometric.

The N–P allometric relationship in the aboveground organs of the different *Calligonum* species showed different trends. For CC, only the ABs were hypoallometric with the scaling exponent = 0.580, while the N–P relationship in the other two organs showed an isometric relationship. For CA, only the mature branches showed an isometric N–P relationship, while the ABs and the reproductive organs exhibited hypoallometric relationships. Furthermore, no significant difference was found in N–P scaling exponents among the three aboveground organs of CA. CK exhibited similar patterns to CC, with only the ABs showing a hypoallometric relationship with the scaling exponent = 0.254. The N–P relationship in the other aboveground organs was isometric. The N–P scaling exponent in the ABs was significantly smaller than in the other two organs. The N–P relationship in all the aboveground organs of CK was isometric.

**Figure 6:**
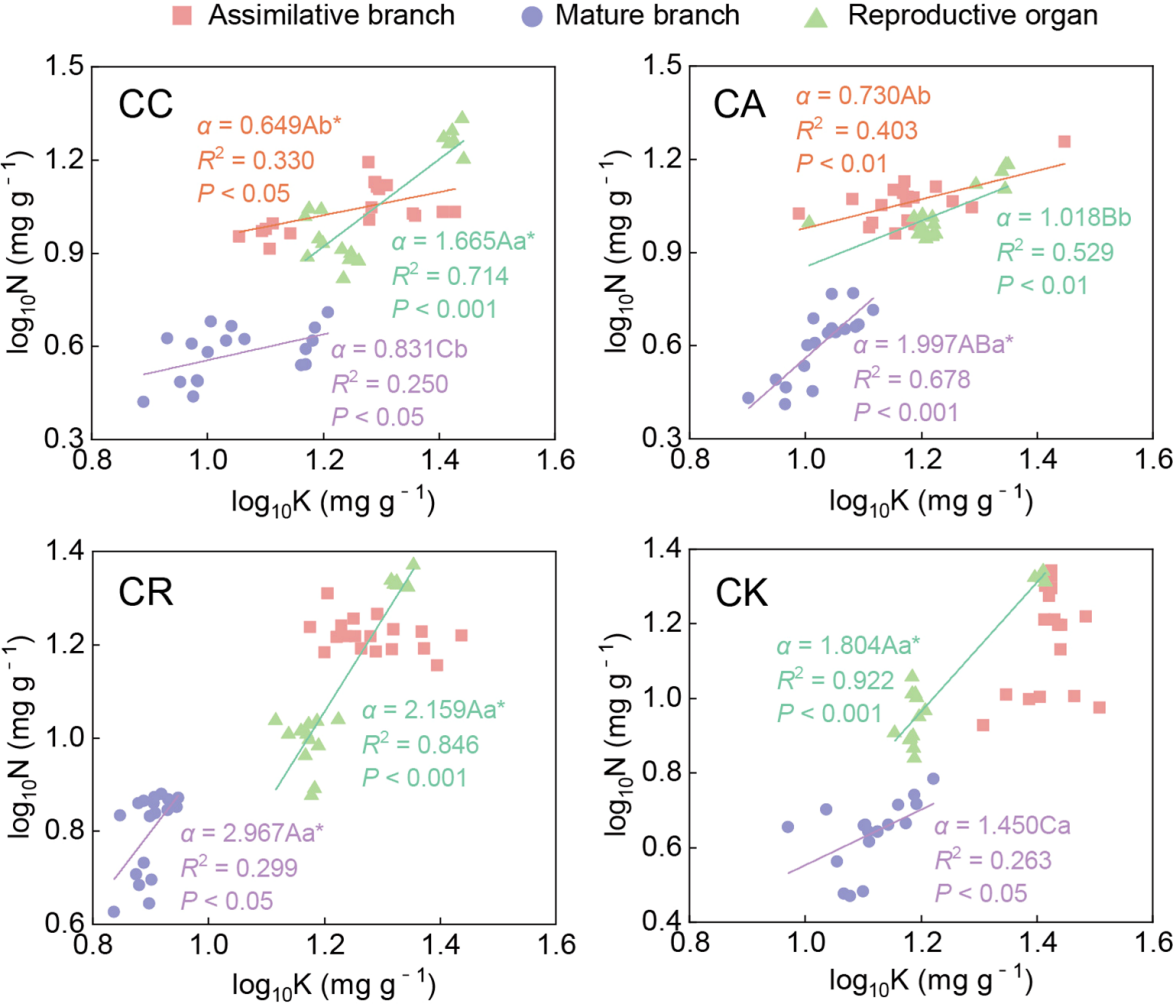
Allometric relationship between N and K in different aboveground organs of four *Calligonum* species. A, B, and C markers for differences between different species for the same organ. a, b, and c markers for differences between different organs of the same species. * marker for significant difference from the assumed slope of 1 (*P* < 0.05).

Analyses of the N–K scaling exponents indicated significant inter-species and organ-specific differences within the four *Calligonum* species (**Figure 6**; **Supplementary Table S1**). For different species, there was no significant difference in N–K exponents between the ABs of CC and CA. The N–K scaling exponent of mature branches showed an isometric relationship between CC and CK; the scaling exponents of CA and CR were significantly greater than 1, among which the scaling exponent of CR (2.967) was the highest, followed by CA (1.997) and CK (1.450); and CC recording the lowest value (0.831). The N–K scaling exponent in the reproductive organs of CA (1.018) was significantly lower than that of the other three species, showing an isometric relationship; and the other three species were all significantly hyperallometric. Differences in the N–K scaling exponent among different aboveground organs of the same species were detected. Among them, the N–K exponent of CR and CK only showed a significant growth relationship in the mature branches and reproductive organs, and no significant difference in the scaling exponent between the aboveground organs of the same species was found. The scaling exponent in the reproductive organs of CC (1.665) was the highest of the N–K scaling exponents among the three aboveground organs. There was no significant difference in the scaling exponent between ABs and mature branches. In contrast, for CA, the N–K scaling exponent in the mature branches (1.997) was significantly higher than that of the other two aboveground organs, and no significant difference in the scaling exponent of the ABs and reproductive organs was detected.

**Figure 7:**
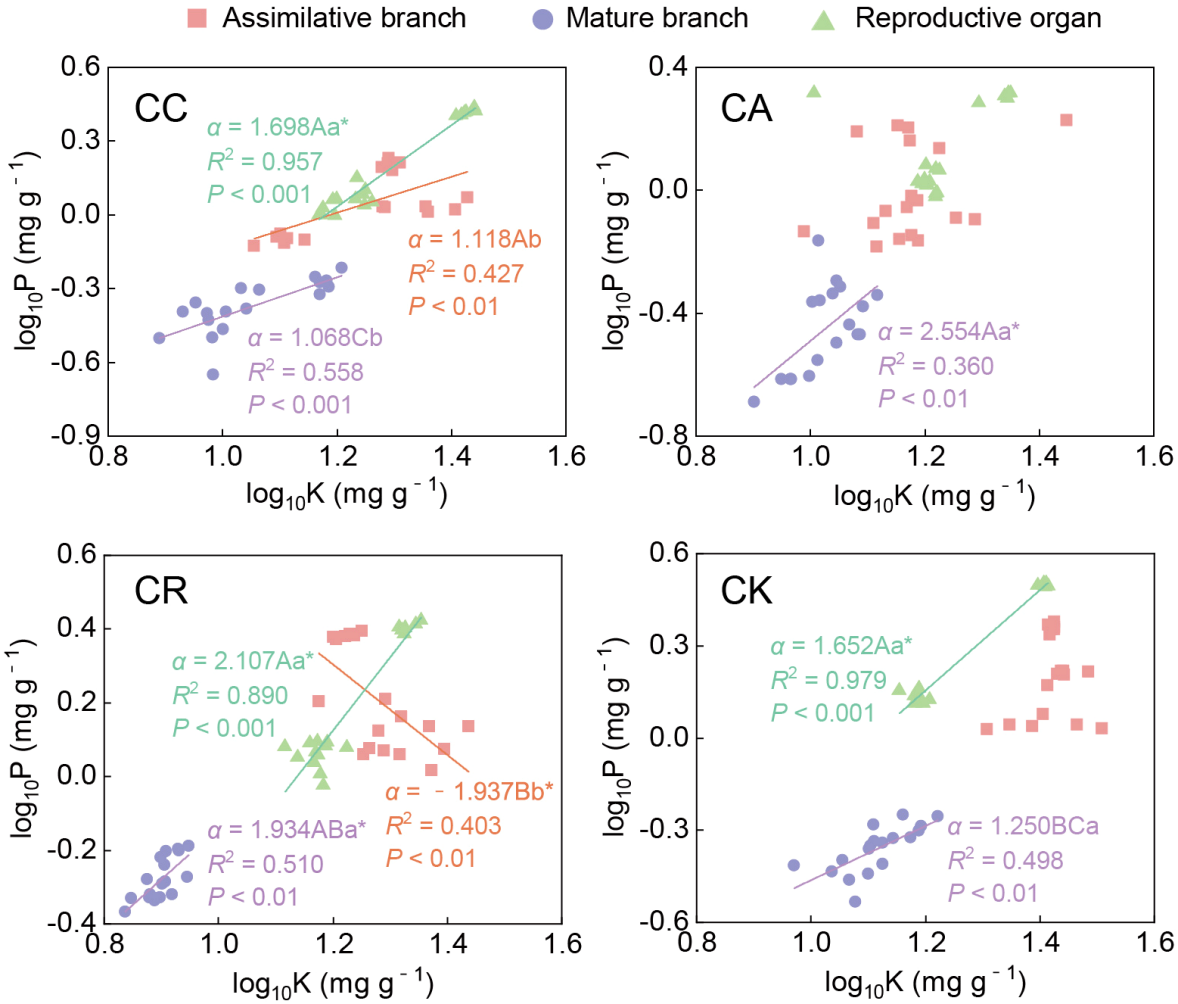
Allometric relationship between P and K in different aboveground organs of four *Calligonum* species. A, B, and C markers for differences between different species for the same organ. a, b, and c markers for differences between different organs of the same species. * marker for significant difference from the assumed slope of 1 (*P* < 0.05).

The P–K scaling exponent also exhibited significant variability among the different species and organs of the four *Calligonum* species (**Figure 7**; **Supplementary Table S1**). The P–K exponent in the ABs of CR was negative (−1.937), which was significantly lower than that of CC (1.118). In mature branches, all the *Calligonum* species had significant P–K allometric relationships. Among them, there was no significant difference between the scaling exponents of CC and CK and 1 (i.e., an isometric relationship). For CA and CR, the scaling exponents were greater than 1, with CA having the highest scaling slope at 2.554. In the reproductive organs, all species had P–K scaling exponents that were significantly greater than 1, without notable inter-species differences. When comparing different aboveground organs, the P–K scaling exponent in the reproductive organs of CC (1.698) was significantly higher than in other aboveground organs. For CR, the scaling slope in the ABs was significantly lower than that in other aboveground organs, and there was no significant difference between the mature branches and reproductive organs. The results of the allometric relationships clearly demonstrated the characteristics and differences of N, P, and K allocation among the different species and aboveground organs and highlighted the important role of unique nutrient allocation strategies in the physiological regulation and stress adaptation of different *Calligonum* species.

## 4 Discussion

### 4.1 Stoichiometric characteristics of different aboveground organs during the reproductive period of desert shrub species with ABs in the common garden

The limiting elements N and P are essential nutrients in the process of plant growth and development. These elements play a crucial role in plant growth and the regulation of various physiological processes, and they are also closely related to each other (Elser et al., 2009; Loladze et al., 2000; Sterner and Hessen, 2003). As an essential mineral element required in significant quantities by plants, K plays a critical role in the process of photosynthesis and the transport of assimilates (Tränkner et al., 2018). In addition, K can also regulate water loss by maintaining cell swelling and osmotic pressure, which is associated with the plant’s inherent drought stress resistance (Battie-Laclau et al., 2014). From the perspective of plant organs, the N, P, and K concentrations in mature branches of the four *Calligonum* species were significantly lower at each reproductive stage. This observation may be attributed to the close relationship between nutrient allocation among different plant aboveground organs and their corresponding functional traits (He et al., 2017). As intermediaries in the transport of water and nutrients, mature branches contain large amounts of lignin and cellulose, which enhance their transport efficiency. Such structural composition can effectively prevent the accumulation of excess N and P in the branches (Zhang et al., 2022). As the substitute for leaves, the ABs were the main organs for photosynthesis, respiration, and transpiration of plants, and their demand for N and P compounds such as proteins and nucleic acids was bound to be high compared with branches (Zhang et al., 2022).

In this study, in the flowering period, the N, P, and K concentrations in the reproductive organs of the four *Calligonum* species were the highest among the three aboveground organs. According to the source-sink relationship hypothesis, during this critical stage of plant growth, nutrients are dynamically transferred from source tissues (such as ABs) to sink tissues (such as developing flowers) to support the rapid development of reproductive organs (Dong and Beckles, 2019). In the flowering period, the reproductive organs of *Calligonum* species serve as the primary nutrient sinks due to their high demand, drawing more N, P, and K to support flower formation and initial fruit development. This nutrient allocation mechanism ensures the efficiency and success of plant reproduction. Yan et al. (2016b) reported a similar result, that is, plants transported greater amounts of N and P to photosynthetic and nutrient-accumulating organs via mature branches. This finding also clarified why N and P concentrations in mature branches were lower than those observed in the ABs and reproductive organs in the present study. Moreover, K also tended to accumulate in the ABs and reproductive organs; this may be because K can promote the diffusion of CO_2_ from the atmosphere into chloroplasts and mediate the distribution of photosynthetic assimilates in plants (Jákli et al., 2016; Tränkner et al., 2018). The ABs and reproductive organs of *Calligonum* species in the common garden exhibited high K concentration across all reproductive stages, which facilitated a high photosynthetic rate during their reproductive period. Similarly, Lu et al. (2016) emphasized that elevated K concentration in the ABs plays a critical role in stabilizing the assimilation rate of plants to maintain high photosynthetic efficiency. Notably, different distribution patterns of N, P, and K among the aboveground organs at different reproductive stages were found. The N and P concentrations in the ABs and reproductive organs, along with the K concentration in the reproductive organs of the four species, were significantly higher than those observed in other developmental stages. This was likely due to plants needing to accumulate large amounts of nutrients before the onset of the flowering period to support flower development (Zheng and Shangguan, 2007), resulting in higher concentrations at this stage. As reproductive activities progressed, nutrients were utilized and consumed, especially N and P in the ABs and the nutrients in the reproductive organs, and thus the nutrients of the ripe-fruit phase were significantly reduced. This could be due to the “reproductive cost”, which leads to reproductive activity under nutrient-limited conditions affecting the nutrient dynamics of plants. Li et al. (2017) supported this perspective through the study on the seasonal nutrient dynamics of *Larix principis-rupprechtii* in plantations. They found that as the plants grew, nutrients were gradually consumed and utilized, resulting in a dilution of nutrient concentrations. As the fruits developed and matured, the demand and consumption of N, P, and K decreased, leading to relatively stable concentrations in the fruits.

Furthermore, the N:P ratio in the flowering phase was significantly lower than in other stages. According to the growth rate hypothesis (Harris, 2003; Niklas and Cobb, 2005), as the relative growth rate increased, N:P showed a decreasing trend and P showed an increasing trend, which indicated that the growth rate of the *Calligonum* species was generally the highest in the three reproductive phases. This result was similar to the study of Dong et al. (2023), which showed that during the reproductive period, plants allocated more nutrients to the reproductive organs to support the high metabolic rate of flowers and seeds. As such, the stoichiometric characteristics were consistent with the growth rate hypothesis to a certain extent. As reproduction continued, it was observed that, except for the N:P ratio of CK, which initially increased before subsequently decreasing, the other three species exhibited a significant increasing trend, indicating a gradual decline in growth rate. This result may be related to the fact that plants ensure their successful reproduction at high temperatures. From April to June, the study area became increasingly hot and arid, and in response to the environmental changes, plants used stress avoidance or adaptation mechanisms to slow down capacity depletion and prolong life by reducing their growth rate (Giorno et al., 2013).

In addition, the N:P threshold hypothesis assumes that there is a specific N:P threshold that can be used to determine the nutrient restriction of plant growth. Koerselman and Meuleman (1996) suggested a threshold range of 14–16 of N:P. It was concluded that when leaves show N:P < 14, the plant growth is limited by N. When N:P > 16, plant growth is limited by P. If the N:P ratio is within this range, plant growth may be co-limited by N and P, or it may not be limited by either nutrient. Güsewell (2004) proposed utilizing the N:P ratios of < 10 and > 20 to determine the type of nutrient limitation of N and P through further fertilization experiments. In this study, the overall N:P ratio was found to be < 14, except for that in the ABs of CA during the ripe-fruit phase, which slightly exceeded 14. This result indicated that the reproductive growth of *Calligonum* in the common garden was primarily limited by N, while the ripe-fruit phase of CA was co-limited by both N and P. Throughout the reproductive period, the N:K ratios in different aboveground organs of the four *Calligonum* species consistently remained < 2.1, while the K:P ratio was > 3.4. According to the standard from Olde Venterink et al. (2003), plants with N:K > 2.1 and K:P < 3.4 are limited by K or N + K. The concentrations and stoichiometric ratios in different aboveground organs of *Calligonum* species displayed interspecific differences, which might be due to the fact that different species respond to environmental changes with different adaptation strategies according to their own properties. This result was indeed consistent with the species composition hypothesis (Reich and Oleksyn, 2004). In summary, this study provides a scientific foundation for understanding nutrient management and optimizing breeding strategies, thereby enhancing our comprehension of the intricate relationship between plant nutrient requirements and physiological functions.

### 4.2 Nutrient covariation relationships among different aboveground organs during the reproductive period of desert shrub species with ABs in the common garden

Through long-term adaptive growth and natural selection, plants have demonstrated varied growth characteristics and nutrient allocation patterns in response to different growth pressures according to their needs (Xiao et al., 2014). The scaling exponent law suggests a power function relationship between the concentrations of all two nutrient elements in plants. This allometric relationship can reveal the basic rates of different nutrient allocations in plants, with the allocation rate reflected by the allometric growth exponent (Niklas et al., 2005; Guo et al., 2019). An isometric relationship typically indicates a constant allocation rate among plant components, whereas an allometric growth relationship highlights an uneven distribution of resources (Xiao et al., 2014). For instance, in the N–P allocation of ABs, only CK exhibited an isometric relationship, whereas the other three species showed a hypoallometric relationship (i.e., slope < 1); among them, the N–P scaling exponent of CR was only 0.254, indicating significant interspecies differences, likely attributable to their unique physiological mechanisms and ecological adaptation strategies. Numerous previous studies have indicated that in accordance with the N–P scaling exponent law, plant resistance is enhanced in high temperature and drought environments, and the levels of resistance-related proteins will increase accordingly, resulting in a higher N–P scaling exponent (generally > 3/4). When integrating leaf N−P scaling exponents from various functional zones, ecological zones, and sampling habitats, these values are observed to approach either 2/3 or 3/4 (Tian et al., 2021; Guo et al., 2020; Priya et al., 2019). Among the four *Calligonum* species, CR exhibited the lowest N–P scaling exponent in the ABs, indicating the least stress and the best adaptation to the common garden environment. CC and CA, introduced from Central Asia, also demonstrated good adaptation (< 3/4). Under drought stress, guard cell K^+^ plays a crucial role in regulating water conductance and transpiration (Chen et al., 2023). For thousands of years, the Turpan Botanical Garden has been exposed to a drought and high-temperature environment. From April to June, the drought and heat stress intensify. The majority of the aboveground organs and species exhibited significantly lower K allocation rates compared to N and P (i.e., the N–K and P–K scaling exponents were significantly less than 1). This indicated that the resistance of the *Calligonum* species to extreme environmental stress in Turpan was generally low; however, interspecies differences were also detected. The result suggested that the nutrient relationships among the different *Calligonum* species still had great heterogeneity. Specifically, CA demonstrated consistent allometric relationships for N–K and P–K in both the ABs and reproductive organs, which may be due to the fact that ABs were the main site for photosynthesis and nutrient accumulation. Moreover, the nutrient utilization of these two aboveground organs remained consistent during the reproductive period, resulting in a uniform nutrient distribution pattern between the ABs and the reproductive organs. This hypothesis was supported by Zhao et al. (2019), who suggested that nutrient allocation among plant organs is often related to the functions and activities of the organs.

Plants allocate nutrients among various organs to meet the specific nutrient demands associated with different functions, thereby facilitating effective reproductive activities. In contrast, mature branches primarily serve supporting and nutrient-transport functions (Liu et al., 2022), resulting in different allometric relationships. In the ABs of CR, the allocation of P and K was negatively correlated, with a scaling exponent of *α* < −1. This indicated that as the allocation rate of K increased, the allocation of P decreased; that is, its resistance increased while its growth rate decreased. Meanwhile, the allocation rates between N and K and between P and K in the mature branches and reproductive organs (with *α* values between 1.934 and 2.967) were significantly greater than 1. That is, the allocation rates of N and P in these organs were 2 to 3 times higher than that of K. This may be due to the fact that during the propagation period, the photosynthesis of the ABs was enhanced by increasing their own K concentration (Tränkner et al., 2018), and the N and P nutrients in the ABs were transferred to the reproductive organs with the assistance of the mature branches, thereby maintaining high N and P allocation rates in both the mature branches and the reproductive organs. The dynamic nutrient transfer and allocation strategy aligned with the concept that plants optimize the allocation of limited resources to enhance overall growth and reproduction (Yu et al., 2015). The result was also similar to the conclusion of many previous studies which showed that plants transfer nutrients among aboveground organs based on their needs, leading to differences in concentrations between different aboveground organs (Li et al., 2017; Yan et al., 2016a). For CK, most nutrient elements in different aboveground organs exhibited an isometric relationship, indicating a relatively consistent demand for N, P, and K across various aboveground organs. However, during the reproductive period, the allocation rates of N and P were generally higher than that of K, suggesting relatively high requirements of N and P for reproductive development. This phenomenon was consistent with the growth rate hypothesis, which posits that faster-growing parts have a more urgent need for nutrients during resource allocation (Dong et al., 2023). This nutrient allocation pattern emphasized CK’s priority strategy for N and P allocation in the reproductive period.

In addition, the N–P scaling exponent in the ABs of CR was significantly lower than that of the other three species (**Supplementary Table S1**), potentially indicating a higher relative growth rate in the reproductive period of CR compared to the others. From **Figure 1**, we also found that the P concentration in the ABs of CR during the flowering phase was significantly higher than that of the other species. This further confirmed its potential to rapidly initiate the growth process. Studies have indicated that plants store a large amount of P in their leaves during the early stages of growth activities, which aids in the synthesis of ribosomal RNA (rRNA), thereby promoting the initiation of growth (Yu et al., 2012). Similarly, Tao et al. (2016) also found that plants in the Karameh region exhibited a high relative growth rate (RGR), as they had higher leaf P concentrations in the leaves. In conclusion, in the reproductive period, different *Calligonum* species demonstrated complex strategies for nutrient allocation and covariation strategies for N, P, and K. These strategies varied among different species, aboveground organs, and stages of the reproductive process. This differentiation reflects the diversity of adaptation to the environment and physiological needs of *Calligonum* species. The heterogeneous allocation of N, P, and K may be related to specific growth requirements, ecological niches, and survival strategies under environmental stress. Understanding the dynamic changes of these nutrients among different species and aboveground organs of *Calligonum* species during various reproductive stages was crucial to revealing how plants regulate their physiological mechanisms to optimize reproductive success and adaptability.

In this study, we thoroughly discussed the nutrient distribution of N, P, and K nutrients in the aboveground organs of different *Calligonum* species at different stages of the reproductive period, and revealed how plants can optimize their reproductive efficiency by finely regulating nutrient allocation, which not only improves our understanding of plant reproductive ecology but also has a profound impact for ecosystem protection and management. The results highlight the importance of maintaining ecosystem biodiversity and stability by optimizing nutrient distribution in plants that improve reproductive efficiency under extreme drought and high temperatures. In the context of global climate change, our research highlights how plants adapt to environmental changes by adjusting their own nutrient allocation, providing new insights into the mechanisms by which ecosystems respond to climate change. In addition, the results provide a scientific basis for future ecological monitoring and research, and for ecosystem protection and management.

## 5 Conclusion

Under common garden conditions, the present study was the first to explore the stoichiometric characteristics of N, P, and K across three aboveground organs (reproductive organs, ABs, and mature branches) of four introduced *Calligonum* species at different reproductive stages. Species, organ, and reproductive stages exhibited significant effects on the nutrient traits of the four species, with organs having the greatest effect. Moreover, significant interactions among the three factors were also detected. Nutrient concentrations in the ABs and reproductive organs were generally higher than those in mature branches. During plant growth, nutrient concentrations gradually decreased, in line with the nutrient dilution effect; however, the N:P ratio in the ABs increased almost entirely, suggesting that their growth rates gradually decreased to adapt to the harsh environment. In the reproductive period, the scaling exponents between N, P, and K in different aboveground organs exhibited interspecies differences, and the differences among the different aboveground organs of each species also existed. These results were consistent with the power law of nutrient relationships and confirmed the species composition hypothesis. Regardless of the organ type, the allocation rate of K was generally lower than that of N and P, indicating that the stress resistance of the four species was generally low. The N–P scaling exponents in the ABs were in the order of CK > CC > CA > CR, indicating that CR had the strongest environmental adaptability and was a species with greater potential for sand fixation. In any case, the growth of *Calligonum* species was primarily limited by N throughout the reproductive period. These results confirmed that in the reproductive period, *Calligonum* species exhibited differentiated nutrient allocation strategies among the different species and aboveground organs in adapting to extreme arid environments, reflecting the complexity and diversity of ecological adaptation.

## Data Availability

The original contributions presented in the study are included in the article/**Supplementary Material**. Further inquiries can be directed to the corresponding author.
