# Supplementary material for: Nutrient allocation patterns in different aboveground organs at different reproductive stages of four introduced Calligonum species in a common garden in northwestern China
Source: Front Plant Sci. 2024 Dec 12;15:1504216. doi: 10.3389/fpls.2024.1504216 (PMC11670320; doi:10.3389/fpls.2024.1504216)
Supplement: Supplementary file 1 [file Table1.docx]

**Table S1**

Allometric relationships among N, P and K in different aboveground organs of four *Calligonum* species

A, B, and C: marker for differences between different species for the same organ. a, b, and c: marker for differences between different organs for the same species.

Calligonum caput-medusae

| *Y* | *X* | Organ | Scaling exponent (*a*) | | | | Isometric test | |
| --- | --- | --- | --- | --- | --- | --- | --- | --- |
|  |  |  | *R*^2^ | *P* | *a* | 95% CI | *F* | *P* |
| log_10_N | log_10_P | Assimilative branches | 0.902 | 0.000 | 0.580Bb | 0.492–0.684 | 53.313 | 0.000 |
|  |  | Mature branches | 0.330 | 0.013 | 0.778Bab | 0.511–1.186 | 1.535 | 0.233 |
|  |  | Reproductive organs | 0.757 | 0.000 | 0.980Aa | 0.757–1.269 | 0.026 | 0.874 |
|  |  | Common slope | – | 0.004 | – | – | – | – |
| log_10_N | log_10_K | Assimilative branches | 0.330 | 0.013 | 0.649Ab | 0.426–0.989 | 4.752 | 0.045 |
|  |  | Mature branches | 0.250 | 0.035 | 0.831Cb | 0.533–1.296 | 0.741 | 0.402 |
|  |  | Reproductive organs | 0.714 | 0.000 | 1.665Aa | 1.258–2.203 | 15.804 | 0.001 |
|  |  | Common slope | – | 0.001 | – | – | – | – |
| log_10_P | log_10_K | Assimilative branches | 0.427 | 0.003 | 1.118Ab | 0.756–1.653 | 0.351 | 0.562 |
|  |  | Mature branches | 0.558 | 0.000 | 1.068Cb | 0.756–1.508 | 0.156 | 0.698 |
|  |  | Reproductive organs | 0.957 | 0.000 | 1.698Aa | 1.522–1.895 | 114.307 | 0.000 |
|  |  | Common slope | – | 0.006 | – | – | – | – |

Calligonum arborescens

| *Y* | *X* | Organ | Scaling exponent (*a*) | | | | Isometric test | |
| --- | --- | --- | --- | --- | --- | --- | --- | --- |
|  |  |  | *R*^2^ | *P* | *a* | 95% CI | *F* | *P* |
| log_10_N | log_10_P | Assimilative branches | 0.618 | 0.000 | 0.459Ba | 0.333–0.634 | 30.932 | 0.000 |
|  |  | Mature branches | 0.474 | 0.002 | 0.782Ba | 0.537–1.138 | 1.878 | 0.189 |
|  |  | Reproductive organs | 0.701 | 0.000 | 0.604Ba | 0.454–0.803 | 14.859 | 0.001 |
|  |  | Common slope | – | 0.092 | 0.588 | 0.484–0.716 | – | – |
| log_10_N | log_10_K | Assimilative branches | 0.403 | 0.005 | 0.730Ab | 0.490–1.088 | 2.742 | 0.117 |
|  |  | Mature branches | 0.678 | 0.000 | 1.997ABa | 1.485–2.686 | 27.843 | 0.000 |
|  |  | Reproductive organs | 0.529 | 0.001 | 1.018Bb | 0.713–1.454 | 0.011 | 0.917 |
|  |  | Common slope | – | 0.001 | – | – | – | – |
| log_10_P | log_10_K | Assimilative branches | – | – | – | – | – | – |
|  |  | Mature branches | 0.360 | 0.008 | 2.554Aa | 1.691–3.856 | 29.221 | 0.000 |
|  |  | Reproductive organs | – | – | – | – | – | – |
|  |  | Common slope | – | 0.261 | 1.952 | 1.489–2.547 | – | – |

Calligonum rubicundum

| *Y* | *X* | Organ | Scaling exponent (*a*) | | | | Isometric test | |
| --- | --- | --- | --- | --- | --- | --- | --- | --- |
|  |  |  | *R*^2^ | *P* | *a* | 95% CI | *F* | *P* |
| log_10_N | log_10_P | Assimilative branches | 0.266 | 0.029 | 0.254Cb | 0.164–0.394 | 74.097 | 0.000 |
|  |  | Mature branches | 0.257 | 0.032 | 1.534Aa | 0.985–2.387 | 4.182 | 0.058 |
|  |  | Reproductive organs | 0.982 | 0.000 | 1.024Aa | 0.954–1.100 | 0.507 | 0.487 |
|  |  | Common slope | – | 0.001 | – | – | – | – |
| log_10_N | log_10_K | Assimilative branches | – | – | – | – | – | – |
|  |  | Mature branches | 0.299 | 0.019 | 2.967Aa | 1.929–4.561 | 39.477 | 0.000 |
|  |  | Reproductive organs | 0.846 | 0.000 | 2.159Aa | 1.756–2.653 | 74.814 | 0.000 |
|  |  | Common slope | – | 0.001 | – | – | – | – |
| log_10_P | log_10_K | Assimilative branches | 0.403 | 0.005 | −1.937Bb | −2.887–−1.300 | 13.516 | 0.002 |
|  |  | Mature branches | 0.510 | 0.001 | 1.934ABa | 1.346–2.781 | 16.400 | 0.001 |
|  |  | Reproductive organs | 0.890 | 0.000 | 2.107Aa | 1.770–2.510 | 97.236 | 0.000 |
|  |  | Common slope | – | 0.863 | 2.056 | 1.775–2.370 | – | – |

Calligonum klementzii

| *Y* | *X* | Organ | Scaling exponent (*a*) | | | | Isometric test | |
| --- | --- | --- | --- | --- | --- | --- | --- | --- |
|  |  |  | *R*^2^ | *P* | *a* | 95% CI | *F* | *P* |
| log_10_N | log_10_P | Assimilative branches | 0.929 | 0.000 | 1.050Aa | 0.912–1.208 | 0.527 | 0.478 |
|  |  | Mature branches | 0.630 | 0.000 | 1.160ABa | 0.845–1.593 | 0.958 | 0.342 |
|  |  | Reproductive organs | 0.903 | 0.000 | 1.092Aa | 0.927–1.287 | 1.286 | 0.274 |
|  |  | Common slope | – | 0.789 | 1.076 | 0.976–1.189 | – | – |
| log_10_N | log_10_K | Assimilative branches | – | – | – | – | – | – |
|  |  | Mature branches | 0.263 | 0.030 | 1.450BCa | 0.933–2.253 | 3.133 | 0.096 |
|  |  | Reproductive organs | 0.922 | 0.000 | 1.804Aa | 1.557–2.090 | 80.442 | 0.000 |
|  |  | Common slope | – | 0.074 | 1.832 | 1.605–2.104 | – | – |
| log_10_P | log_10_K | Assimilative branches | – | – | – | – | – | – |
|  |  | Mature branches | 0.498 | 0.001 | 1.250BCa | 0.866–1.804 | 1.612 | 0.222 |
|  |  | Reproductive organs | 0.979 | 0.000 | 1.652Aa | 1.529–1.785 | 205.083 | 0.000 |
|  |  | Common slope | – | 0.021 | – | – | – | – |

**Table S2**

Monthly average values of the main meteorological factors in the three sampling periods in the study area (Turpan Desert Botanical Garden).

| Time | Average monthly temperature  (℃) | Average monthly humidity  (%) | | Direct radiation  (W/m²) | Photosynthetically active radiation  (W/m²) | Surface temperature  (℃) | Daily maximum temperature  (℃) |
| --- | --- | --- | --- | --- | --- | --- | --- |
| April | 21.278 | | 20.335 | 200.781 | 301.437 | 27.146 | 29.209 |
| May | 28.520 | | 22.944 | 261.007 | 484.204 | 37.113 | 35.857 |
| June | 30.358 | | 29.927 | 262.474 | 516.364 | 39.821 | 37.561 |
